# A case of poorly differentiated adenocarcinoma with lymphoid stroma originated in the ascending colon diagnosed as lymphoepithelioma-like carcinoma

**DOI:** 10.1007/s12328-019-01081-8

**Published:** 2019-12-16

**Authors:** Kengo Kai, Hideki Hidaka, Takeshi Nakamura, Yuji Ueda, Kosuke Marutsuka, Takuto Ikeda, Atsushi Nanashima

**Affiliations:** 1 Department of Surgery, Miyazaki Prefectural Miyazaki Hospital, 5-30 Kitatakamatsu, Miyazaki City, Miyazaki 8808510 Japan; 2 Department of Pathology, Miyazaki Prefectural Miyazaki Hospital, 5-30 Kitatakamatsu, Miyazaki City, Miyazaki 8808510 Japan; 3 Department of Surgery, Faculty of Medicine, University of Miyazaki, 5200 Kihara, Kiyotake, Miyazaki City, Miyazaki 8891692 Japan

**Keywords:** Carcinoma with lymphoid stroma, Ascending colon cancer, Lymphoepithelioma-like carcinoma, Medullary carcinoma, EBER-ISH (EBV-encoded small RNA-in situ hybridization)

## Abstract

An 86-year-old woman’s stool sample was positive for blood. Computed tomography (CT) showed wall thickening of the ascending colon at the hepatic flexure. Colonoscopy showed near-complete obturation by colon cancer. Since she was asymptomatic, elective surgery was planned. Laparoscopic right hemicolectomy was performed. Histopathological examination showed poorly differentiated carcinoma cells proliferating in a solid pattern with marked lymphocyte infiltration. The diagnosis was lymphoepithelioma-like carcinoma (LELC) associated with Epstein-Barr virus (EBV) infection; however, EBV-encoded small RNA–in situ hybridization was negative. Microsatellite instability was not assessed. The postoperative course was uneventful and she was discharged on the 15th postoperative day. She remains recurrence-free at 2 years after surgery. Past reports note that colorectal carcinomas with dense lymphoid stroma may be related to LELC or medullary carcinoma (MC). Gastrointestinal LELC is rare, with some reports on LELC of the esophagus and stomach. Reports on LELC of the large intestine are very rare. MC of the large intestine is relatively new concept, firstly described in the WHO Classification of Tumours of the Digestive System 3rd Edition in 2000. We herein present a case of lymphoepithelioma-like carcinoma of the ascending colon and relevant case reports about LELC and MC of the large intestine.

## Introduction

Poorly differentiated adenocarcinoma associated with dense lymphoid stroma may be associated with two subsets: lymphoepithelioma-like carcinoma (LELC), which is associated with Epstein Barr virus (EBV) infection; and medullary carcinoma (MC), which is associated with high microsatellite instability (MSI-H) [1]. LELC occurring in the gastrointestinal tract is rare, with some reports describing the involvement of the esophagus and stomach. Reports on colon involvement are very scarce. MC of the large intestine was first described in the World Health Organization Classification of Tumours of the digestive system 3rd Edition in 2000 [2]. Reported cases are uncommon, similarly to LELC of the large intestine. Both types are associated with a good prognosis in comparison to poorly differentiated colorectal carcinoma. We herein report a case of poorly differentiated adenocarcinoma with lymphoid stroma of the ascending colon that was diagnosed as LELC based on the similarity to LELC involving other organs, such as stomach or lung. We also review past case reports on LELC and MC of the large intestine.

## Case report

**Fig. 1:**
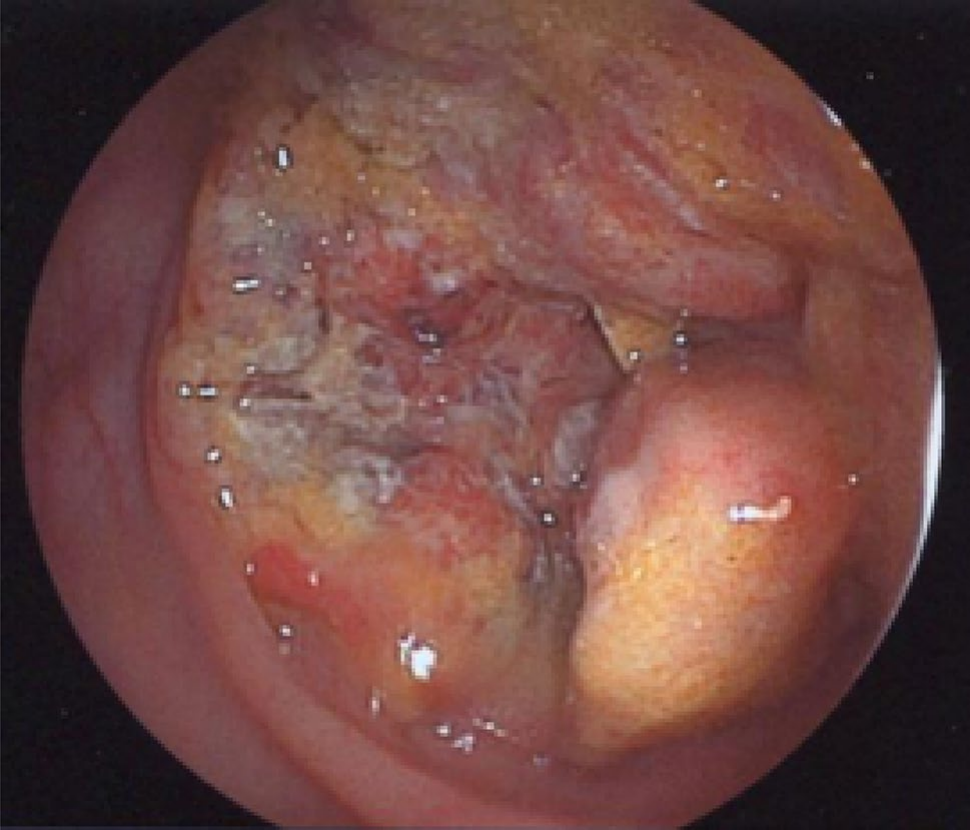
Colonoscopy: circumferential cancer located in the ascending colon almost obturated the colonic lumen and the scope could not pass through the tumor to the oral side

**Fig. 2:**
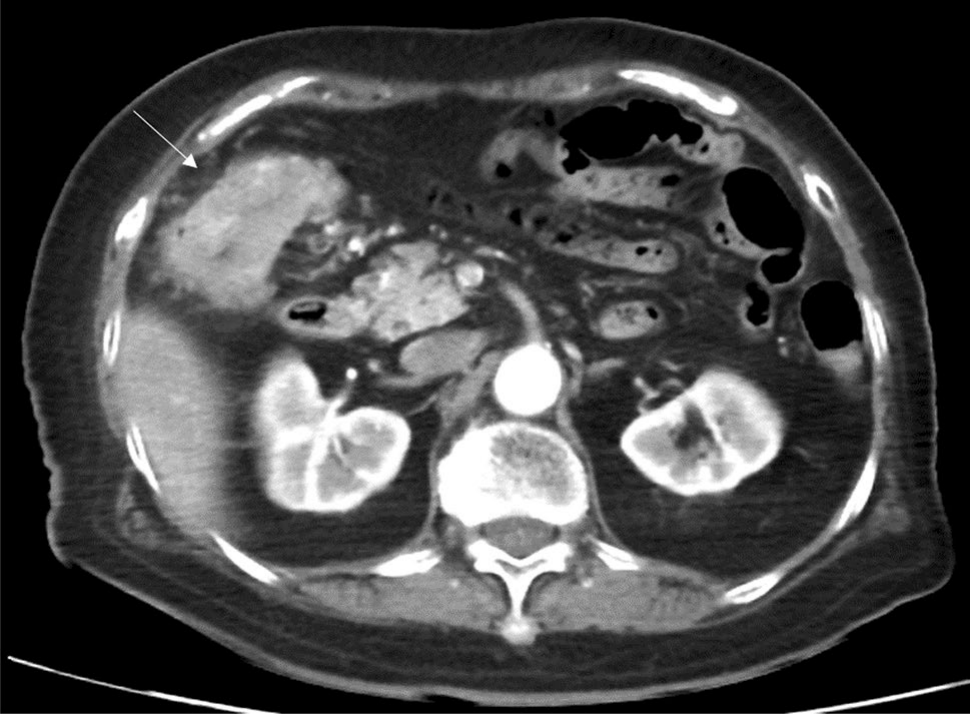
Enhanced computed tomography: wall thickening with enhancement at the hepatic flexure (arrow) without the findings suggesting lymph node metastasis, distant metastasis and intestinal bowel obstruction

The patient was an 86-year-old woman who was initially managed by a general practitioner. She underwent colonoscopy after blood was detected in a stool sample. Colonoscopy revealed circumferential cancer in the ascending colon. The colonic lumen was almost obturated and the scope could not pass through the tumor to the oral side (Fig. 1). The patient was transferred to our hospital for surgical treatment. She did not have a family history and past history of malignant disease. The patient was not in good health and had been diagnosed with dementia and disuse syndrome after lumber compression fracture. An abdominal examination revealed a palpable mass without tenderness. With the exception of anemia (hemoglobin, 11.7 g/dL [normal range: 13.2–17.2 g/dL]), the patient’s laboratory findings ware unremarkable, including tumor marker levels such as carcinoembryonic antigen (CEA), carbohydrate antigen 19–9 (CA19-9). Enhanced computed tomography (CT) revealed wall thickening with the enhancement of the ascending colon at the hepatic flexure without the findings suggesting lymph node metastasis, distant metastasis or intestinal obstruction (Fig. 2).

These findings led to a diagnosis of advanced ascending colon cancer of T3N0M0 cStage IIA, according to the International Union Against Cancer TNM Classification(UICC), 8th edition. Since she had no symptoms, elective surgery was planned for 12 days after admission. Laparoscopic right hemicolectomy and D3 lymph node dissection were performed. Ileocolonic anastomosis was reconstructed with functional end-to-end anastomosis technique.

**Fig. 3:**
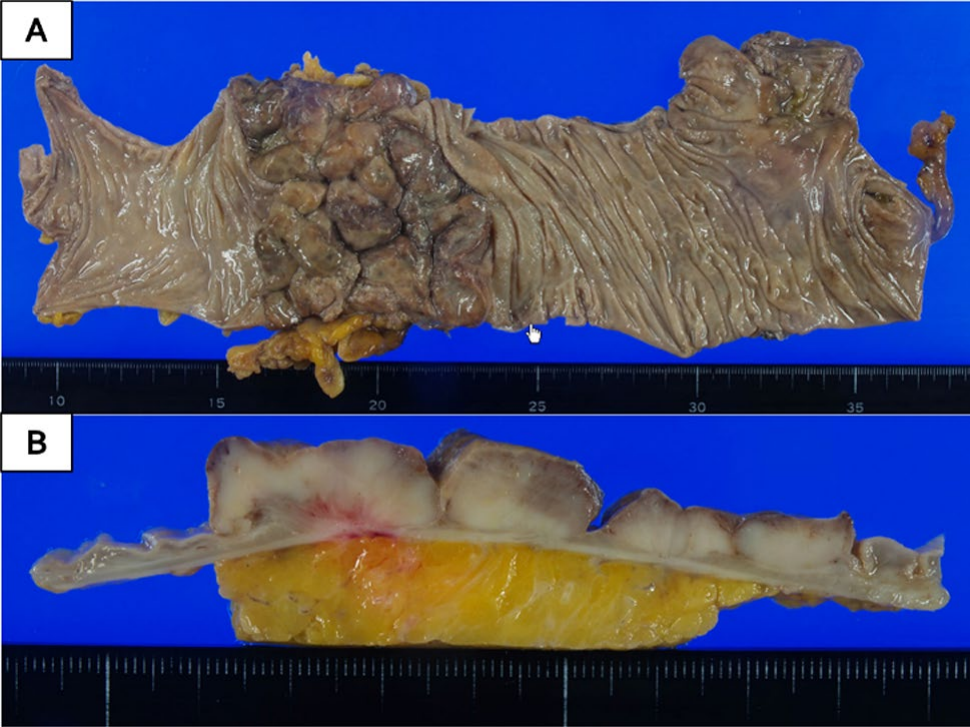
Macroscopic examination: The resected specimen showed type 1 cancer resembling like a nodule-aggregated lesion, which measured 75 × 63 mm in size (**a**, **b**)

**Fig. 4:**
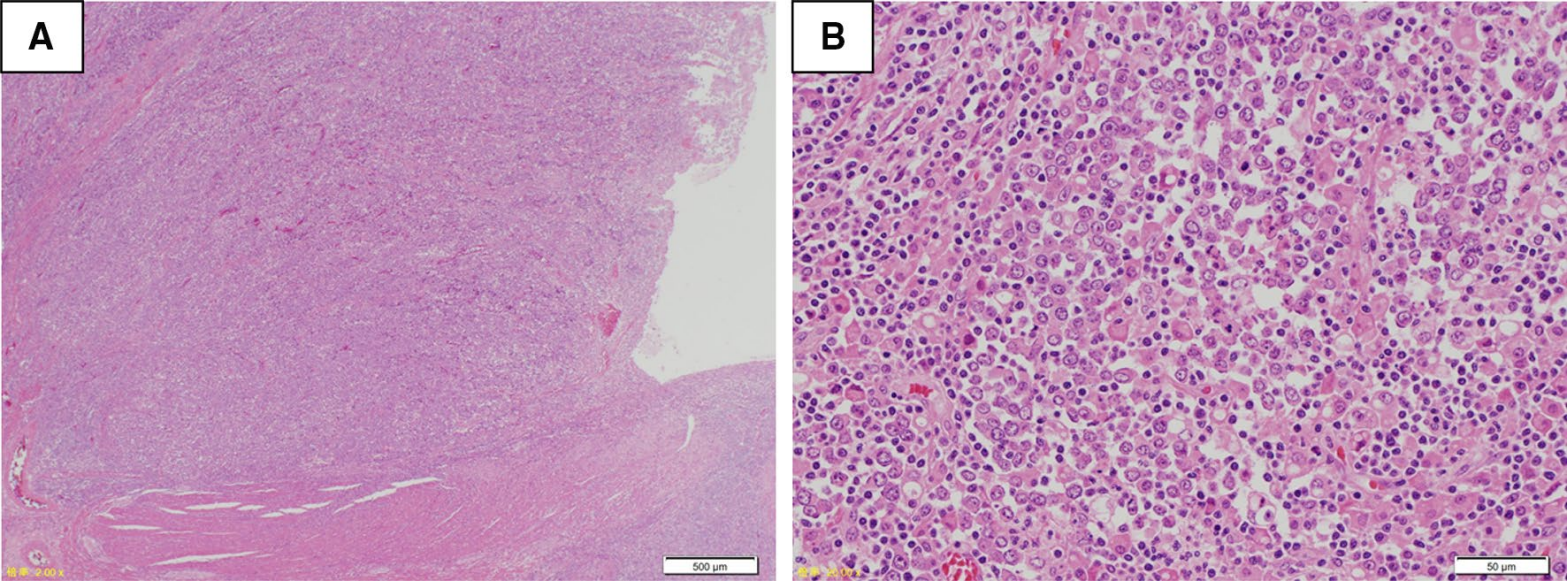
Microscopic findings (Hematoxylin and eosin stain): In low power view, tumor shows nodular growth, occupying the entire wall (**a**). In the high power view, the undifferentiated tumor cells with large pleomorphic nuclei and some small nucleoli proliferate diffusely without cohesiveness, accompanied by diffuse lymphoplasmacytic infiltration in the tumor (**b**)

**Fig. 5:**
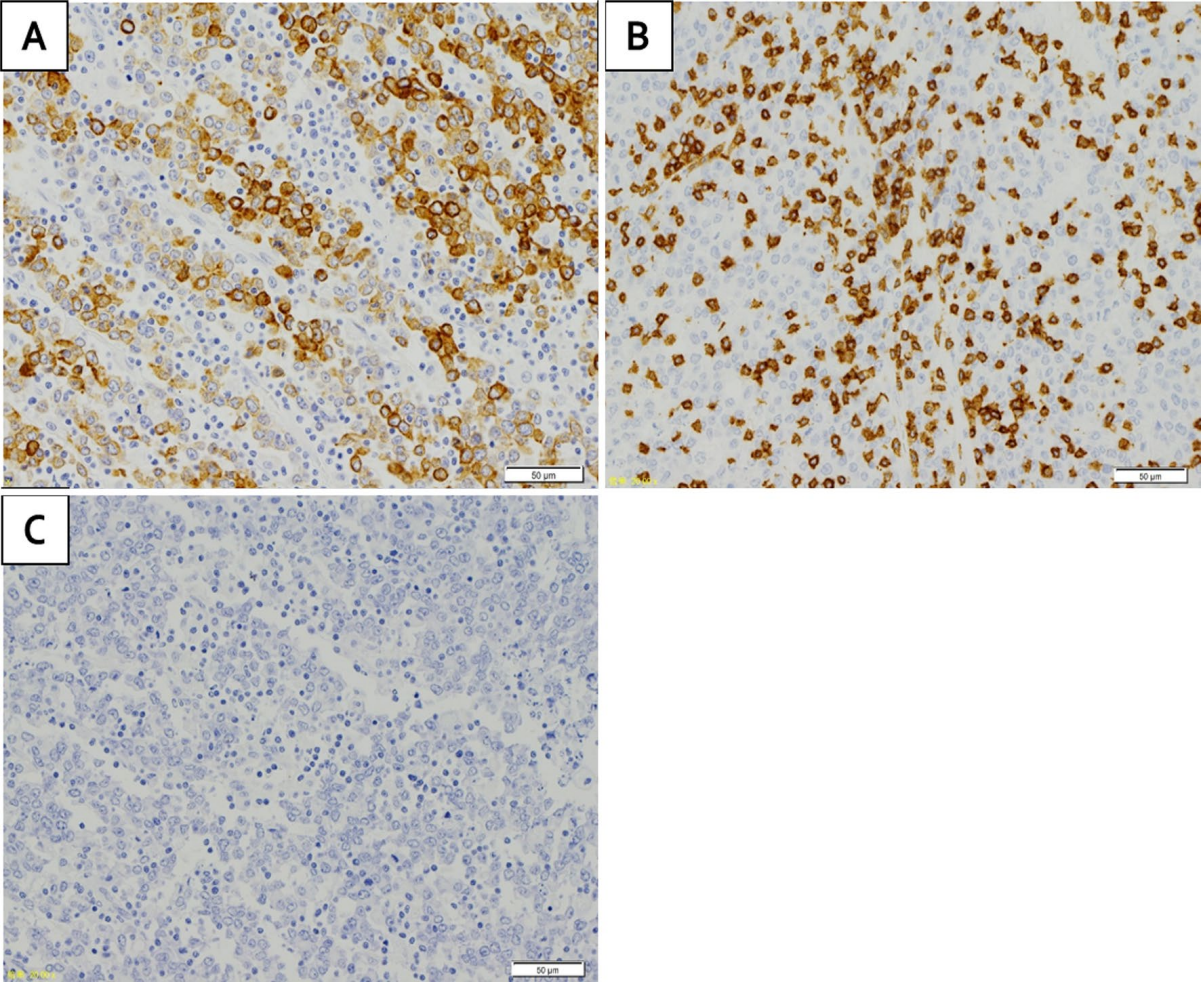
Pathologic findings (Immunohistochemical staining): **a** positive staining for AE1/AE3 in LELC. **b** Positive staining for CD3 in the lymphocytes. EBV-encoded small RNA-in situ hybridization (EBER-ISH) revealed that tumor cells and lymphocytes were negative for EBV

The resected specimen revealed a nodule-aggregated lesion of 75 × 63 mm in size (Fig. 3). Hematoxylin and eosin staining showed that tumor demonstrated nodular growth, occupying the entire wall in low power view (Fig. 4a) and that the undifferentiated tumor cells with large pleomorphic nuclei and some small nucleoli proliferate diffusely without cohesiveness, accompanied by diffuse lymphoplasmacytic infiltration in the tumor in high power view (Fig. 4b). Immunostaining revealed to be predominantly composed of T-lymphocytes (Fig. 5b). Thus, it was believed to be a primary colorectal LELC with dense lymphoid stroma. The tumor exhibited no lymphatic invasion and was positive for por1, pSS, int, INFc, ly2, v1, PM0, DM0, RM0. An immunohistological assessment of the tumor cells using EBV-encoded small RNA- in situ hybridization was negative for EBV (Fig. 5c).

The patient’s postoperative course was uneventful and she was discharged on the 15th postoperative day. No evidence of cancer recurrence has been seen during 2 years of follow-up without adjuvant chemotherapy after the surgery.

## Discussion

We described a case of poorly differentiated adenocarcinoma with lymphoid stroma originated in the ascending colon that was diagnosed as LELC. The term “lymphoepithelioma” was coined in 1921 by Regaud and Reverchon [3] and Schminke [4] to describe a tumor occurring in the nasopharynx, related to EBV infection. This tumor is characteristically composed of cells with large vesicular nuclei, single prominent nucleoli, and indistinct cell borders, which impart a syncytial appearance to the tumor, and has an attendant lymphoplasmacytic inflammatory component [5]. With this hallmark, LELC has been described at various locations outside the nasopharynx, including the thymus, salivary gland, lung, vagina, skin, urinary bladder and mammary gland; however, the majority of these cases were not associated with EBV. LELC occurring in the gastrointestinal tract is uncommon, and the best recognized example of LELC in the gastrointestinal tract occurs in the stomach. LELC of the stomach was first described by Watanabe et al. in 1976 as “gastric carcinoma with a lymphoid stroma” [6]. EBV is associated with 5–20% of gastric carcinomas worldwide [7–9]; however, more than 80% of LELCs of the stomach have been found to be related to EBV infection [10] in comparison to only approximately 6–7% of non-LELC [11].

With regards to colorectal LELC, thus far only 10 cases have been reported, including our case [12–20] (Table 1). According to previous case reports, LELC of the large intestine is diagnosed based on the pathological similarity to LELC of other organ. The tumor cells are large, oval or polygonal in shape, contain vesicular to clear nuclei, prominent nucleoli, and abundant eosinophilic cytoplasm with poorly defined cell borders [21, 22]. The small lymphocytes can infiltrate cancer cell nests and epithelioid granulomas are sometimes observed within the lymphoid stroma. The lymphoid reaction is composed predominantly of T-cells, including cytotoxic lymphocytes and/or natural killer cells in close contact with tumor cells [23]. The mechanism by which EBV contributes to the lymphocytic reaction for LELC is still unknown. Poorly differentiated adenocarcinoma is the most frequent type of LELC. However, LELC is known to be relatively less aggressive (clinically), despite its poorly differentiated features [24].

**Table 1:** Reported cases of lymphoepithelioma-like carcinoma of the large intestine including our case (10 cases)

Case no	Author	Year	Age	Sex	Site	Tumor size (mm)	T	N	M	MSI assesment	EBER-ISH in tumor cells
1	Vilor [12]	1995	77	F	T	120	3	1	0	Not described	Negative
2	Palazzo [13]	1995	29	M	R	35	3	–	–	Not described	Negative
3	Samaha [14]	1998	62	M	C	28	3	1	–	Not described	Not described
4	De Petris [15]	1999	44	M	A	9	1	–	–	Not performed (HNPCC)	Negative
5	Kon [16]	2001	72	M	R	25	2	0	–	Not described	Positive
6	Kojima [17]	2006	25	M	R	7	1	0	0	Not described	Weak positive
7	Taniguchi [18]	2011	88	F	T	40	3	0	–	Not described	Negative
8	Delaney [19]	2012	85	F	S	42	2	0	–	Loss of MLH-1 and PMS-2	Negative
9	Mori [20]	2013	70	F	A	70	1	0	–	Not described	Negative
10	Our case	2019	86	F	T	75	3	0	0	Not performed	Negative

*MSI* microsatellite instability, *EBER-ISH* EBV-encoded small RNA–in situ hybridization, *HNPCC* hereditary non-polyposis colorectal cancer

The other type of the colorectal cancer with dense lymphoid stroma is medullary carcinoma (MC). Colorectal MC is a rare disease, only 10 cases reported in the literature to date; colorectal MC is almost always associated with a microsatellites instability-high (MSI-H) status [25–33] (Table 2). Thirunavukarasu et al. [34] reported that MC was a rare tumor, accounting for approximately 5–8 of every 10,000 colon cancers diagnosed. Similarly to LELC, MC should be distinguished from other more aggressive colorectal cancers because MC appears to be associated with better survival outcomes than undifferentiated colon adenocarcinoma. Patients with MC commonly present with Stage II disease, with 10% presenting metastasis. An analysis of the early outcomes in patients with MC revealed that 1- and 2- year relative survival rates of 92.7% and 73.8%, respectively [34]. MC is morphologically characterized by sheets of malignant cells with vesicular nuclei, prominent nucleoli and abundant eosinophilic cytoplasm, as well as the prominent infiltration of tumoral lymphocytes. MC is known to be associated with MSI-H, and some studies examining the immunohistochemical characteristics of this tumor have shown that it can be differentiated from poorly differentiated colorectal adenocarcinoma by a loss of MLH-1 staining and the intestinal transcription factor CDX2.

**Table 2:** Reported cases of medullary carcinoma of the large intestine (10 cases)

Case no	Author	Year	Age	Sex	Site	Tumor size (mm)	T	N	M	MSI-H assess ment	EBER-ISH in tumor cell
1	Wang [25]	2005	79	F	A	90	–	1	0	Not described	Not described
2	Sakurai [26]	2012	78	F	T	20	1	0	0	Loss of MLH-1 and PMS-2	Not described
3	Cunningham [27]	2014	79	F	A	60	3	1	1	Loss of MLH-1 and PMS-2	Not described
4	Cunningham [27]	2014	81	F	T	–	4	0	0	Loss of MLH-1 and PMS-2	Not described
5	Mitchell [28]	2014	75	F	C	40	2	2	0	Loss of MLH-1 and PMS-2	Negative
6	Jain [29]	2014	72	F	D	80	2	0	0	Loss of MLH-1 and PMS-2	Not described
7	Kasapidis [30]	2015	58	M	A	–	3	1	0	Not described	Not described
8	Wakasugi [31]	2017	72	F	T	60	3	0	0	Loss of MLH-1 and CDX-2	Not described
9	Martinotti [32]	2017	44	F	A	60	3	0	0	Loss of MLH-1 and CDX-2	Negative
10	Yago [33]	2018	63	M	A	75	2	0	0	Loss of MLH-1	Not described

*MSI* microsatellite instability, *EBER-ISH* EBV-encoded small RNA–in situ hybridization

Colorectal cancer with MSI-H is classified into two subtype; hereditary non-polyposis colorectal cancer (HNPCC) typically occurs in the youth, while sporadic colorectal cancer with MSI-I typically occurs in the elderly [35]. The mean age at the diagnosis of MC of the large intestine is reported to be 69 years, with the incidence increasing with age [34]. HNPCC is characterized by an autosomal dominant inherited pattern of susceptibility to colorectal cancer at an early age, the multiplicity of colorectal cancer, proximal tumor location and potential carcinogenesis of the endometrium and other organs [36]. HNPCC is diagnosed based on stringent clinical criteria (the Amsterdam criteria) and germline mutations in mismatch repair genes associated with MSI-H, such as MLH1, MSH2, PMS1 and PMS2 [37]. Interestingly, De Petris et al. [15] reported a case of LELC of the colon in a patient with HNPCC. From the description of the tumor, it would appear to conform to the morphological criteria for an LELC rather than an MC. Furthermore, the patient was negative for EBV infection. Because of the family history and the pathologic feature, the patient was believed to be affected by HNPCC. The diagnosis of HNPCC was confirmed in a center for hereditary gastrointestinal cancer; however, the patient refused to undergo genetic testing.

In a review article, Chetty et al. [1] noted that MC demonstrates a syncytial growth pattern, bears robust peritumoral inflammation, and has a well-defined border, while LELC is formed by small clusters of cells that “do not correspond to the syncytial growth patterns seen in MC”, has more prominent intratumoral than peritumoral inflammation, and has an infiltrative border. On the other hand, Gonzalez et al. [38] reported that these tumors are often classified together, thereby implying that they are the same entity. They do not recommend relying on histologic criteria alone to distinguish between LELC and MC, because of the overlap in syncytial and clustered architecture and the fact that intratumoral and peritumoral inflammation are not mutually exclusive. Thus, they report that the term, “carcinoma with lymphoid stroma”, should best serve as a categorizing diagnosis after the initial histological review. Determining the EBV and MSI status via ancillary testing currently appears to be the most reliable way to arrive at a final diagnosis.

In this case, the pathologist in our institution diagnosed poorly differentiated carcinoma with lymphoid stroma with LELC based on morphologic features, which were similar to LELC of other organs, especially in terms of intratumoral-dominant inflammation and an undefined border. While we considered performing an additional assessment to determine the MSI status, we did not perform the assessment because the possibility of HNPCC was considered to be very low based on the patient’s age and family history. Of course, it is interesting to concern the pathogenesis of the carcinoma with molecular status, but it seems that it be more important to keep in mind the possibility of HNPCC in colon carcinoma with lymphoid stroma and to interview the patient based on Amsterdam criteria according to the age. The past cases suggest that when a physician encounters colon carcinoma with a dense lymphoid stroma, it is clinically important to perform an assessment for HNPCC, regardless of whether LELC or MC is diagnosed based on the histological findings.
